# The safety and efficacy of vortioxetine for acute treatment of major depressive disorder: a systematic review and meta-analysis

**DOI:** 10.1186/s13643-015-0001-y

**Published:** 2015-03-01

**Authors:** Amanda S Meeker, Megan C Herink, Dean G Haxby, Daniel M Hartung

**Affiliations:** Department of Pharmacy Practice, College of Pharmacy, Oregon State University/Oregon Health and Science University, 2730 SW Moody Ave, Mail code: CL5CP, Portland, OR 97239 USA

**Keywords:** Antidepressant, Depression, Systematic review, Meta-analysis

## Abstract

**Background:**

Vortioxetine is the first mixed serotonin agonist and antagonist antidepressant approved in the US. We sought to evaluate all published and unpublished data available to determine the efficacy and harms of vortioxetine in adults with major depressive disorder.

**Methods:**

We used a predefined search strategy of MEDLINE, the Cochrane Central Register of Controlled Trials, ClinicalTrials.gov, and Drugs@FDA to identify studies evaluating vortioxetine in the acute treatment of major depressive disorder. Only randomized controlled trials (RCTs) that provided results on relevant clinical efficacy and safety outcomes were included. Study quality was assessed and results were pooled using mixed effect meta-analyses where applicable.

**Results:**

We identified 11 RCTs with 6,145 participants meeting inclusion criteria (eight were published and three were unpublished). The trials did not exceed 8 weeks in duration. The response rate with vortioxetine was significantly higher for 1-mg (relative risk (RR) = 1.91; 95% confidence interval (CI) 1.36 to 2.69), 5-mg (RR = 1.33; 95% CI 1.10 to 1.61), 10-mg (RR = 1.42; 95% CI 1.21 to 1.67), and 20-mg doses (RR = 1.58; 95% CI 1.19 to 2.08) compared to placebo. Remission rates were significantly higher for the 10-mg group (RR = 1.45; 95% CI 1.18 to 1.77) and the 20-mg group (RR = 1.68; 95% CI 1.19 to 2.37) compared to placebo. Meta-regression of dose on the log odds ratio of response was not statistically significant (*β* = 0.01; *P* = 0.46). Vortioxetine response rates were lower than active serotonin and norepinephrine reuptake inhibitor (SNRI) comparators for the 5-mg (RR = 0.88; 95% CI 0.80 to 0.98), 15-mg (RR = 0.78; 95% CI 0.68 to 0.90), and 20-mg (RR = 0.82; 95% CI 0.72 to 0.94) doses. The most common adverse events were nausea and vomiting which increased in frequency with higher doses.

**Conclusions:**

Vortioxetine was significantly more effective than placebo for acute treatment of major depressive disorder (MDD). Although treatment effect estimates varied substantially between studies, a dose effect was not observed. Vortioxetine does not appear to be more effective, and is potentially less effective, than an SNRI.

**Systematic review registration:**

PROSPERO CRD42013006198.

**Electronic supplementary material:**

The online version of this article (doi:10.1186/s13643-015-0001-y) contains supplementary material, which is available to authorized users.

## Background

Depressive disorders, including major depressive disorder (MDD), are common mental health conditions thought to be caused by an imbalance in serotonin (5-HT) and norepinephrine in addition to multiple situational, cognitive, and medical factors. Pharmacotherapy is commonly used in the medical management of depressive disorders and may include first-generation antidepressants (tricyclic antidepressants and monoamine oxidase inhibitors) and second-generation antidepressants (selective serotonin reuptake inhibitors (SSRIs) and serotonin and norepinephrine reuptake inhibitors (SNRIs)). These drugs selectively modulate neurotransmitters, including 5-HT, norepinephrine, and dopamine, in the central nervous system.

Current evidence suggests that most second-generation antidepressants have similar efficacy for the treatment of MDD [1,2]. SSRIs are often recommended as first-line therapy because they have a favorable risk-benefit ratio compared to first-generation antidepressants and SNRIs [3]. In October 2013, the US Food and Drug Administration (FDA) approved vortioxetine for the treatment of MMD [4]. Vortioxetine has also been approved by the European Medicines Agency (EMA). Different than other approved SSRIs, vortioxetine is a multimodal antidepressant believed to work through a mix of 5-HT agonism and antagonism. To date, seven distinct families of 5-HT receptors have been identified (5-HT_1_ to 5-HT_7_) and subpopulations have been described for 5-HT_1_ and 5-HT_2_. Antidepressant activity is mediated through agonism at 5-HT_1A_, 5-HT_1B_, 5-HT_2C_, 5-HT_4_, and 5-HT_6_, as well as antagonism at 5-HT_2A_, 5-HT_3_, and 5-HT_7_ [5]. *In vitro* data suggest vortioxetine is an antagonist of 5-HT_3_, 5-HT_7_, and 5-HT_1D_; an agonist of 5-HT_1A_; a partial agonist of 5-HT_1B_; and an inhibitor of 5-HT transporter [6].

According to the FDA label, the efficacy of vortioxetine was demonstrated in six positive randomized clinical trials (RCTs) of 6- to 8-weeks duration. In their review, the FDA deemed the 20-mg dose as the most consistently positive dosing arm. They therefore advise that patients should be started on 10 mg/day, and the dosage should be increased to 20 mg/day as tolerated. Because publication bias is a well-recognized concern in the antidepressant literature, it is unclear if these six trials represent the entire trial program for vortioxetine [7]. To address this uncertainty, we conducted a systematic review of published and unpublished data to summarize the efficacy and harms of vortioxetine for the treatment of MDD.

## Methods

For this systematic review, we considered the following questions: 1) for patients with acute MDD, what is the efficacy of vortioxetine compared to other antidepressants or placebo; 2) for patients with acute MDD, what are the harms of vortioxetine compared with other antidepressants or placebo. A structured protocol was developed *a priori* (PROSPERO Registration ID: CRD42013006198). To identify relevant articles, we conducted a focused Medline and EMBASE search through 18 September 2014 using the following terms: (vortioxetine) OR (Lu AA21004). Supplemental searches were conducted using the Cochrane Central Register of Controlled Trials using analogous terms. We also identified relevant studies through a review of ClinicalTrials.gov, the FDA website (Drugs@FDA), and requested trial information from the manufacturer (Takeda). The citations of yielded articles were reviewed to identify other potentially relevant studies.

### Study selection

RCTs investigating the safety and efficacy of vortioxetine for acute treatment of MDD compared to placebo or another antidepressant were included. Only original research studies that provided results on relevant clinical outcomes in a useable format were included. There were no limits on race, ethnicities, cultural groups, language, or setting. Editorials, letters, and non-systematic literature reviews were not included. Results from our search were reviewed independently by two investigators (ASM and DMH). Discrepancies were resolved through consensus.

### Outcome measures

The primary efficacy outcomes were response and remission. Response is typically defined by a decrease of ≥50% in the Hamilton Depression Rating Scale (HAMD) or Montgomery-Åsberg Depression Rating Scale (MADRS) scores from baseline [8]. Remission is defined by a total HAMD (≤7) or MADRS (≤10) score [8]. Secondary efficacy outcomes of interest were absolute change in HAMD and MADRS scores from baseline. We also summarized the rates of serious adverse events, common adverse events (>5%) including nausea, diarrhea, dry mouth, and withdrawal due to adverse events.

### Data extraction

One author (ASM) extracted trial data into evidence tables describing the population characteristics, study subject selection and attrition, primary efficacy, and harm findings; a second author (DMH) validated the data. Our primary sources of data were publications. For dichotomous outcomes, we used the reported denominator for each outcome. We also attempted to obtain data from unpublished trials or trials with insufficiently reported outcomes through examination of ClinicalTrials.gov, Drugs@FDA, and the manufacturers’ dossier. ClinicalTrials.gov is the largest publically accessible clinical trial registry in the world. As of 2008, certain trials of drugs, biologics, and devices regulated by the US FDA are required to report summary results data in ClinicalTrials.gov within 1 year of trial completion. ClinicalTrials.gov’s required reporting elements include basic demographics, all primary and secondary outcomes, and adverse events.

### Quality assessment

To assess the quality of studies, we used predefined criteria based on those developed by the Pacific Northwest Evidence-Based Practice Center Drug Effectiveness Review Project (DERP) [9]. In general, a “good” study has the least bias and results are considered to be valid, a “fair” study is susceptible to some bias but probably not sufficient to invalidate its results and a “poor” rating indicates significant bias that may invalidate the study’s results. Three members of the team (DMH, MCH, and ASM) independently reviewed included papers using the DERP criteria and assigned each study an overall quality rating. Conflicts were resolved by discussion and consensus. Unpublished studies were assessed for quality on evaluable characteristics (baseline similarity among treatment groups, attrition, and use of an intention-to-treat protocol).

### Statistical analysis

Quantitative synthesis of outcomes was performed using random effects meta-analysis. Risk ratios (relative risk (RRs)) and 95% confidence intervals (95% confidence intervals (CIs)) were calculated for dichotomous outcomes of interest (e.g., response rates). A random effects model using the DerSimonian and Laird method was used to calculate for the weighted mean effect size for trials by dosing arm. The *I*^2^ statistic was calculated to describe the proportion of the variability that was due to heterogeneity rather than sampling error. We explored observed heterogeneity quantitatively and qualitatively using meta-regression and sensitivity analysis. Publication bias was evaluated using a funnel plot and Egger’s test. Stata13™ (Stat Corp; College Station, TX) was used to carry out all statistical analyses [10]. The findings of this systematic review are reported according to the guidelines of the Preferred Reporting Items for Systematic Reviews and Meta-Analyses (PRISMA) [11].

## Results

**Figure 1:**
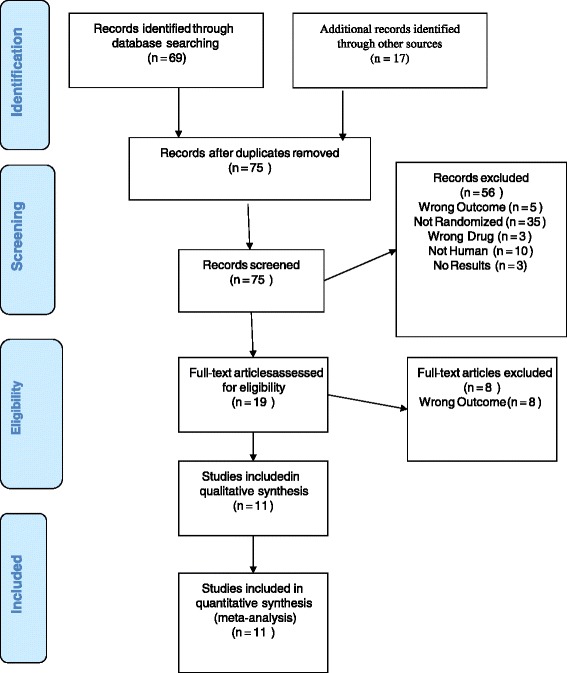
**PRISMA flowchart of study selection.**

The literature search resulted in a total of 75 records after duplicates were removed. Of these, 56 were excluded because they did not meet inclusion criteria, and 19 candidate trials were assessed for eligibility. Eleven remaining RCTs with a total of 6,145 patients fulfilled the inclusion criteria (Figure 1). Of these 11 trials, eight were in peer-reviewed publications and three were unpublished but had results summarized in FDA review documents or ClinicalTrials.gov. Table 1 summarizes the characteristics and findings of the included studies. Trials ranged in size from 429 to 776 participants. All trials were placebo controlled; six trials also included an active comparator arm with an SNRI (one trial included venlafaxine and five trials included duloxetine). Several trials studied more than one vortioxetine dose ranging from 1 to 20 mg. Five trials were conducted within the US, four were outside the US, and two included US and non-US sites. Of the published trials, all but one were rated fair or good quality. The trial by Henigsberg et al [12] was determined to be of poor quality because it did not use an intent-to-treat analysis and several critical trial features were not clearly described. All unpublished studies were rated fair quality based on evaluable domains (intention-to-treat analysis, similar baseline characteristics, attrition). Details of trial findings and quality appraisal can be found in Additional file 1.

**Table 1:** **Summary of included trials**

**Author NCT identifier FDA identifier**	**Country**	***N***	**Arms**	**Mean age**	**Baseline MADRS score**	**% non-white**	**% female**	**Follow-up**	**Study quality**
Alvarez et al NCT00839423 11492A^a^	Non-US	429	V 5 V 10 mg VLFX 225 mg	43.3	34.0	8.0	62.7	6 weeks	Good
Mahableshwarker et al NCT00672620 304^b^	US	611	V 2.5 mg V 5 mg DLX 60 mg placebo	42.7	29.8	26.0	63.5	8 weeks	Fair
Baldwin et al NCT00635219 11984A^b^	Non-US	776	V 2.5 mg V 5 mg V 10 mg DLX 60 mg placebo	44.9	31.9	21	68.1	8 weeks	Fair
Jain et al NCT00672958 303^b^	US	600	V 5 mg placebo	42.4	34.1	29.2	58.3	6 weeks	Fair
Katona et al NCT00811252 12541A^a^	US and Non-US	453	V 5 mg DLX 60 mg placebo	70.6	30.5	5.3	65.7	8 weeks	Good
Henigsberg et alNCT00735709 305^a^	Non-US	560	V 1 mg V 5 mg V 10 mg placebo	46.4	30.8	13.8	62.7	8 weeks	Poor
Boulenger et al NCT01140906 13267A^a^	Non-US	608	V 15 mg V 20 mg DLX 60 mg placebo	46.7	31.4	1.8	65.9	8 weeks	Fair
McIntyre et al NCT01422213 FOCUS	US and Non-US	598	V 10 mg V 20 mg placebo	45.7	31.5	5.5	66.2	8 weeks	Good
Unpublished NCT01153009 315^a^	US	614	V 15 mg V 20 mg DLX 60 mg placebo	42.9	32.1	23.4	73.8	8 weeks	Fair^c^
Unpublished NCT01163266 316^a^	US	462	V 10 mg V 20 mg placebo	42.8	32.2	30.1	72.5	8 weeks	Fair^c^
Unpublished NCT01179516 317^b^	US	434	V 10 mg V 15 mg placebo	45.1	33.7	25.8	70.1	8 weeks	Fair^c^

V = vortioxetine; VLFX = venlafaxine XR; DLX = duloxetine; NR = not reported; NCT = national clinical trial number. ^a^Considered positive by FDA; ^b^considered failed or negative by FDA; ^c^only evaluable trial characteristics assessed.

### Efficacy compared to placebo

**Figure 2:**
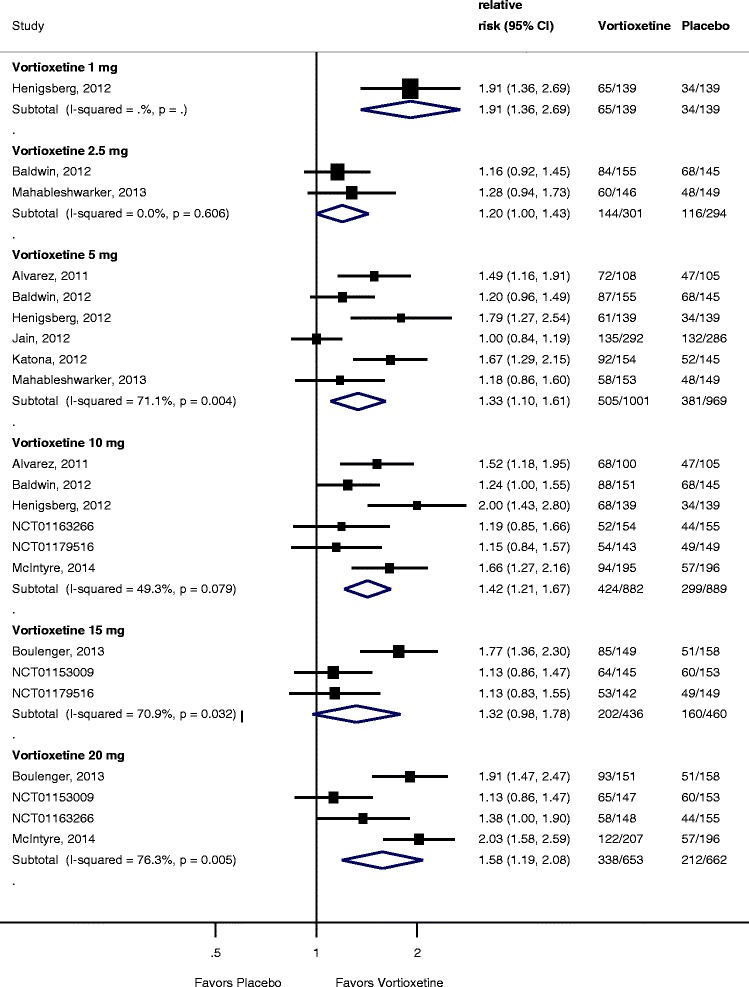
**Forest plot showing response rates for vortioxetine by dose compared to placebo.**

Trials used either or both of the MADRS or HAMD scales to measure efficacy of vortioxetine. Nine trials compared vortioxetine to placebo for response using the MADRS scale at 6 to 8 weeks [6,12-16]. Two trials defined response using the HAMD scale [17,18]. Three trials assessed response with both scales [6,12,14]. For our analysis of response, we first synthesized the MADRS response using HAMD response for the two trials not measuring MADRS response. As shown in Figure 2, compared to placebo, response rates were significantly higher for vortioxetine 1-mg (RR = 1.91; 95% CI 1.36 to 2.69; *I*^2^ = not applicable), 5-mg (RR = 1.33; 95% CI 1.10 to 1.61; *I*^2^ = 71%), 10-mg (RR = 1.42; 95% CI 1.21 to 1.67; *I*^2^ = 49.3%), and 20-mg dose groups (RR = 1.58; 95% CI 1.19 to 2.08; *I*^2^ = 76.3%). Heterogeneity was very high for most of the dose comparisons. Removing the two trials that did not measure response using MADRS had no meaningful impact on the RRs for response at any dosing level.

**Figure 3:**
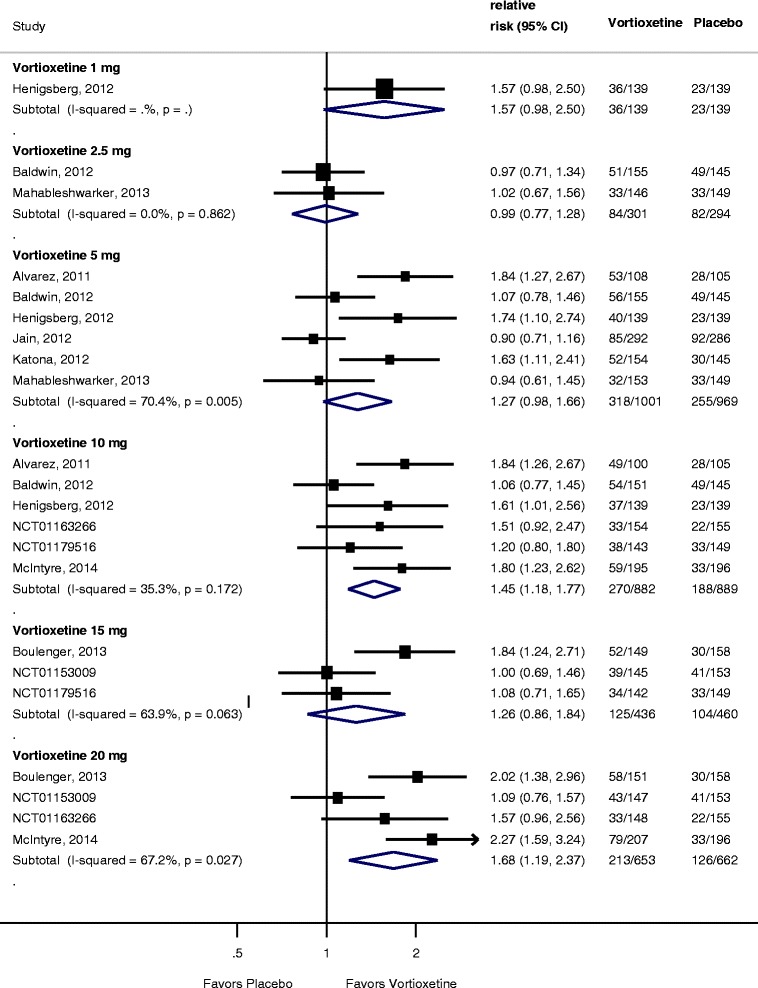
**Forest plot showing remission rates for vortioxetine by dose compared to placebo.**

All 11 trials compared vortioxetine to placebo in remission based on the MADRS scale. The meta-analysis of remission is summarized in Figure 3. Results demonstrated a statistically significant difference in remission rates for the 10-mg (RR 1.45; 95% CI 1.18 to 1.77; *I*^2^ = 35%) and the 20-mg groups (RR 1.68; 95% CI 1.19 to 2.37; *I*^2^ = 67%) compared to placebo, but no difference for the 1-mg (1.57; 95% CI 0.98 to 2.50; *I*^2^ = not applicable), 2.5-mg (RR 0.99; 95% CI 0.77 to 1.28; *I*^2^ = 0%), 5-mg (RR 1.27; 95% CI 0.98 to 1.66; *I*^2^ = 70.4%), and 15-mg groups (RR 1.26; 95% CI 0.86 to 1.84; *I*^2^ = 63.9%) compared to placebo. There was considerable heterogeneity between trials for each dose.

**Figure 4:**
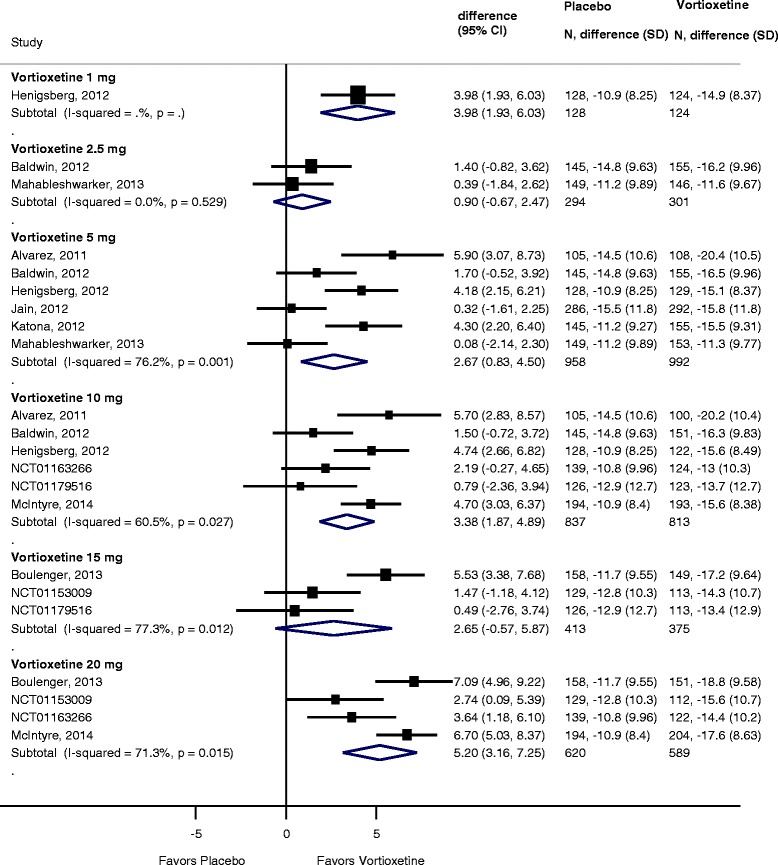
**Forest plot showing change from baseline in MADRS score for vortioxetine by dose compared to placebo.**

All 11 trials reported changes from baseline in the MADRS total score. Figure 4 summarizes the synthesis of these data by dosing level compared to placebo. Significant reductions in MADRS compared to placebo ranged from 2.67 (95% CI 0.83 to 4.5; *I*^2^ = 76%) for the 5-mg dose to 5.20 (95% CI 3.16 to 7.25; *I*^2^ = 71.3%) for the 20-mg dose. There was no significant difference in change from baseline in the 2.5 or 15-mg dose groups. Similar to response and remission, heterogeneity was very high.

**Figure 5:**
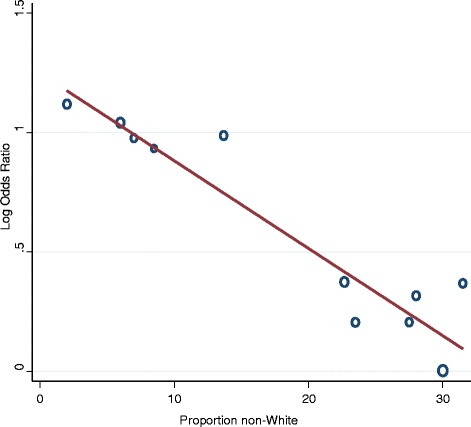
**Meta-regression plot showing relationship between proportion of non-White study participants and the log odds ratio for response.** Vortioxetine dose arms combined. *β* coefficient = −0.04 (95% CI −0.05 to −0.02).

Table 2 summarizes meta-regressions performed in an attempt to identify and explain trial heterogeneity. We first examined the association between dose and response. Nine trials had more than one vortioxetine dosing arm. For this meta-regression, we partitioned the number of responders and denominator from the placebo arm equally among the vortioxetine arms to avoid double counting subjects receiving placebo [19]. For example, in the trial by Alvarez et al. [6], 47 out of 105 subjects receiving placebo had a response. Because the trial had two vortioxetine arms, we compared each active treatment arm (5 and 10 mg) to a placebo group of 52.5 participants, of which 23.5 responded. Meta-regression of dose on the log odds ratio of response was not statistically significant (*β* = 0.01; *P* = 0.46). Meta-regression of dose on absolute change in MADRS, without placebo arm partitioning, was also not significant (*β* = 0.13; *P* = 0.09). Because of an apparent lack of dose response, we pooled the dosing arms for the remaining meta-regressions. Study quality, publication status, or a combination of both (poor quality or unpublished) had no impact on response. The only variables significantly associated with response were whether or not the study was conducted in the US (*β* = −0.7; *P* = 0.001) and the proportion of study participants who were not White (*β* = −0.04; *P* < 0.001). The later variable resulted in 0% residual variation due to heterogeneity and is graphically depicted in Figure 5. Re-analyzing the studies by the proportion of non-White participants (>20% non-White, ≤20% non-White) eliminated nearly all statistical heterogeneity between studies.

**Table 2:** **Meta-regression of study characteristics on log odds ratio for response**

**Variable**	**Coefficient**	**95% confidence interval**	***P*** **value**	**Residual** ***I*** ^**2**^
Dose^a^	0.01	−0.02 to 0.04	0.46	67%
Unpublished	−0.44	−1.04 to 0.16	0.13	74%
Poor quality	0.44	−0.57 to 1.45	0.35	77%
Poor quality or unpublished study	−0.23	−0.84 to 0.38	0.42	78%
Non-US-based study	−0.70	−1.02 to -0.35	0.001	24%
Duration 8 weeks (vs 6 weeks)	0.10	−0.29 to 0.48	0.58	77%
Baseline MADRS score	−0.11	−0.33 to 0.11	0.29	75%
Proportion of study participants non-White	−0.04	−0.05 to -0.02	<0.001	0%

^a^Placebo arm partitioned for multi-arm trial.

### Adverse events

Table 3 summarizes pooled adverse event (AE) absolute risk differences for each vortioxetine dose compared to placebo. The most frequently reported AEs were nausea and vomiting. At the 20-mg dose, 20.3% (95% CI 16.5% to 24.2%) and 5.5% (95% CI 1.2% to 9.8%) more patients treated with vortioxetine than placebo experienced nausea and vomiting, respectively. The frequency of nausea in the one trial evaluating the 1-mg dose (risk difference = 3.6%; 95% −2.0% to 9.2%) was similar to placebo, suggesting there is an increase in nausea as the dose increases. Withdrawals due to an AE were significantly more common than placebo at the 10-, 15-, and 20-mg doses, but not significantly different for the 1-, 2.5-, and 5-mg doses. There were no differences in the incidence of serious adverse events.

**Table 3:** **Absolute risk difference of adverse events for vortioxetine compared to placebo**

	**1 mg**	**2.5 mg**	**5 mg**	**10 mg**	**15 mg**	**20 mg**
Withdrawals due to adverse events	0.7% (−2.4% to 3.8%) 1 trial	−0.6% (−4.3% to 3.1%); *I* ^2^ = 0% 2 trials	0.1% (−1.5% to 1.6%); *I* ^2^ = 1.6% 6 trials	1.9% (0.1% to 3.7%)*; *I* ^2^ = 0% 6 trials	4.4% (1.4% to 7.4%)*; *I* ^2^ = 0% 3 trials	3.6% (0.1% to 7.2%)*; *I* ^2^ = 58% 4 trials
Serious adverse events	−0.7% (−3.1% to 1.7%) 1 trial	−1.3% (−3% to 0.3%); *I* ^2^ = 0% 2 trials	0% (−1% to 1%); *I* ^2^ = 0% 6 trials	0% (−0.9% to 0.9%); *I* ^2^ = 0% 6 trials	0.1% (−0.9% to 1.0%); *I* ^2^ = 2% 3 trials	0.1% (−0.6% to 0.9%); *I* ^2^ = 0% 4 trials
Nausea	3.6% (−2.0% to 9.2%) 1 trial	6.8% (1.4% to 12.1%)*; *I* ^2^ = 0% 2 trials	12.4% (8.9% to 15.9%)*; *I* ^2^ = 23% 6 trials	16.6% (11.1% to 22.2%)*; *I* ^2^ = 68% 6 trials	21.0% (15.9% to 26.1%)*; *I* ^2^ = 0% 3 trials	20.3% (16.5% to 24.2%)*; *I* ^2^ = 0% 4 trials
Vomiting	not reported	0.1% (−1.6% to 1.8%); *I* ^2^ = 0% 2 trials	1.6% (0.1% to 3.1%)*; *I* ^2^ = 0% 4 trials	3.3% (0.8% to 5.9%)*; *I* ^2^ = 20% 4 trials	6.4% (0.7% to 12.1%)*; *I* ^2^ = 65% 2 trials	5.5% (1.2% to 9.8%)*; *I* ^2^ = 49% 2 trials
Headache	−1.4% (−7.5% to 4.6%) 1 trial	−0.1% (−5.6% to 5.4%); *I* ^2^ = 0% 2 trials	−0.5% (−4.2% to 3.2%); *I* ^2^ = 30% 6 trials	−0.5% (−3.3% to 2.4%); *I* ^2^ = 0% 6 trials	2.2% (−2.1% to 6.5%); *I* ^2^ = 0% 3 trials	4.1% (−0.7% to 7.5%); *I* ^2^ = 0% 4 trials
Diarrhea	0% (−2.8% to 2.8%) 1 trial	−3.0% (−6.8% to 0.8%); *I* ^2^ = 0% 2 trials	0.3% (−2.5% to 3.1%); *I* ^2^ = 48% 6 trials	1.1% (−1.1% to 3.3%); *I* ^2^ = 0% 5 trials	3.7% (−1.5% to 8.8%); *I* ^2^ = 60% 3 trials	1.4% (−1.8% to 4.7%); *I* ^2^ = 0% 3 trials
Dizziness	−1.4% (−4.2% to 1.3%) 1 trial	−0.1% (−4.3% to 4.1%); *I* ^2^ = 24% 2 trials	−0.1% (−2.1% to 1.8%); *I* ^2^ = 0% 6 trials	−0.3% (−4.0% to 3.4%); *I* ^2^ = 56% 5 trials	1.3% (−4.4% to 7.0%); *I* ^2^ = 68% 3 trials	2.9% (−3.6% to 9.4%); *I* ^2^ = 76% 3 trials
Dry mouth	−0.7% (−3.8% to 2.4%) 1 trial	−0.3% (−7.3% to 6.7%); *I* ^2^ = 65% 2 trials	0.8% (−1.2% to 2.8%); *I* ^2^ = 0% 6 trials	−0.1% (−4.1% to 4.0%); *I* ^2^ = 64% 5 trials	−0.5% (−3.3% to 2.4%); *I* ^2^ = 0% 3 trials	0.6% (−4.9% to 6.1%); *I* ^2^ = 63% 3 trials
Hyperhydrosis	−0.7% (−2.7% to 1.3%) 1 trial	−0.0% (−1.6% to 1.6%); *I* ^2^ = 0% 2 trials	0.5% (−0.7% to 1.8%); *I* ^2^ = 0% 6 trials	2.4% (−1.2% to 6.0%); *I* ^2^ = 71% 3 trials	−1.0% (−3.2% to 1.1%); *I* ^2^ = 0% 2 trials	−1.8% (−5.6% to 2.0%); *I* ^2^ = 66% 2 trials
Nasopharyngitis	−2.1% (−7.1% to 2.8%) 1 trial	3.7% (−1.6% to 9.0%) 1 trial	0.7% (−1.2% to 2.5%); *I* ^2^ = 0% 4 trials	−0.7% (−2.5% to 1.2%); *I* ^2^ = 15% 6 trials	−2.7% (−5.8% to 0.4%); *I* ^2^ = 0% 2 trials	1.2% (−2.2% to 4.6%); *I* ^2^ = 48% 3 trials
Insomnia	not reported	0.5% (−2.7% to 3.8%); *I* ^2^ = 0% 2 trials	−0.0% (−1.9% to 1.9%); *I* ^2^ = 0% 4 trials	−0.4% (−2.5% to 1.7%); *I* ^2^ = 0% 4 trials	0.1% (−2.9% to 3.2%); *I* ^2^ = 5% 2 trials	0.4% (−5.1% to 5.9%); *I* ^2^ = 64% 2 trials
Fatigue	2.1% (−0.6% to 4.9%) 1 trial	−1.4% (−3.5% to 0.8%); *I* ^2^ = 0% 2 trials	0.4% (−1.4% to 2.2%); *I* ^2^ = 34% 6 trials	0.2% (−1.2% to 1.7%); *I* ^2^ = 0% 5 trials	0.5% (−2.2% to 3.2%); *I* ^2^ = 24% 3 trials	−0.8% (−5.8% to 4.2%); *I* ^2^ = 79% 3 trials

*P < 0.05.

### Comparative efficacy

**Figure 6:**
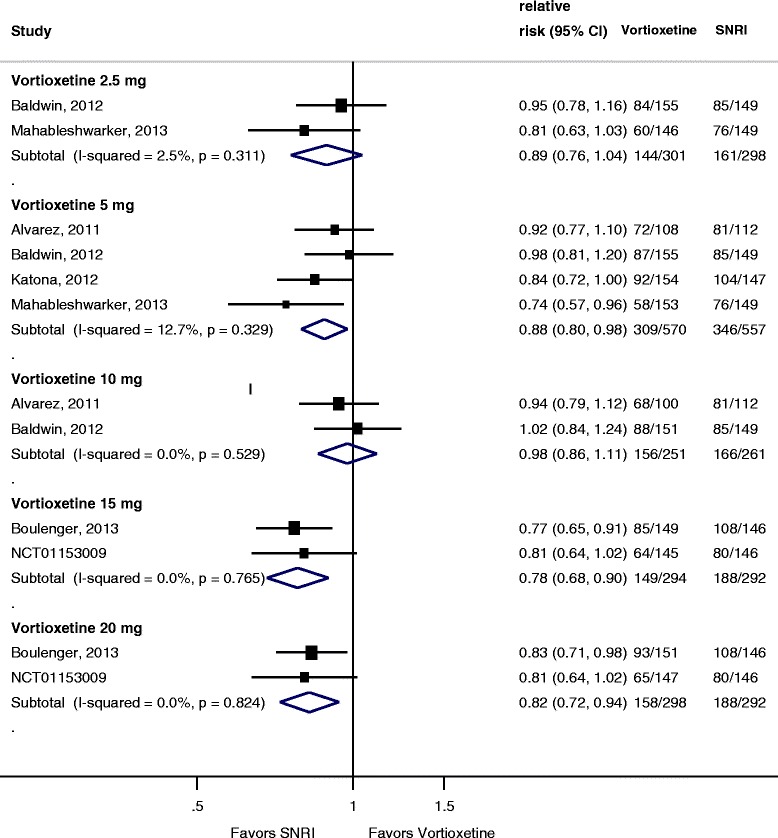
**Forest plot showing response rates for vortioxetine by dose compared to a serotonin norepinephrine reuptake inhibitor.**

**Figure 7:**
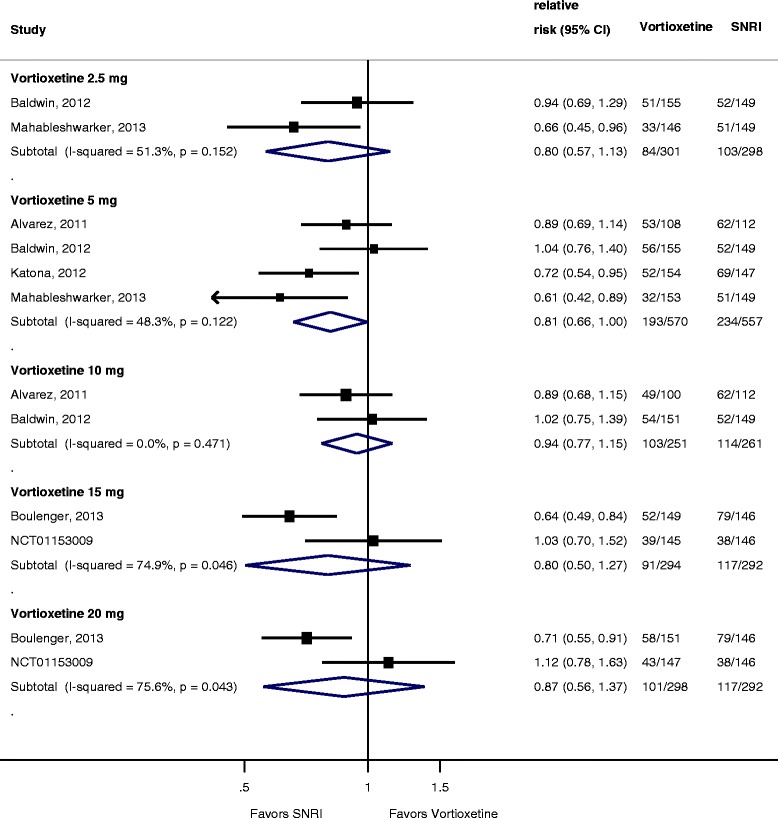
**Forest plot showing remission rates for vortioxetine by dose compared to a serotonin norepinephrine reuptake inhibitor.**

**Figure 8:**
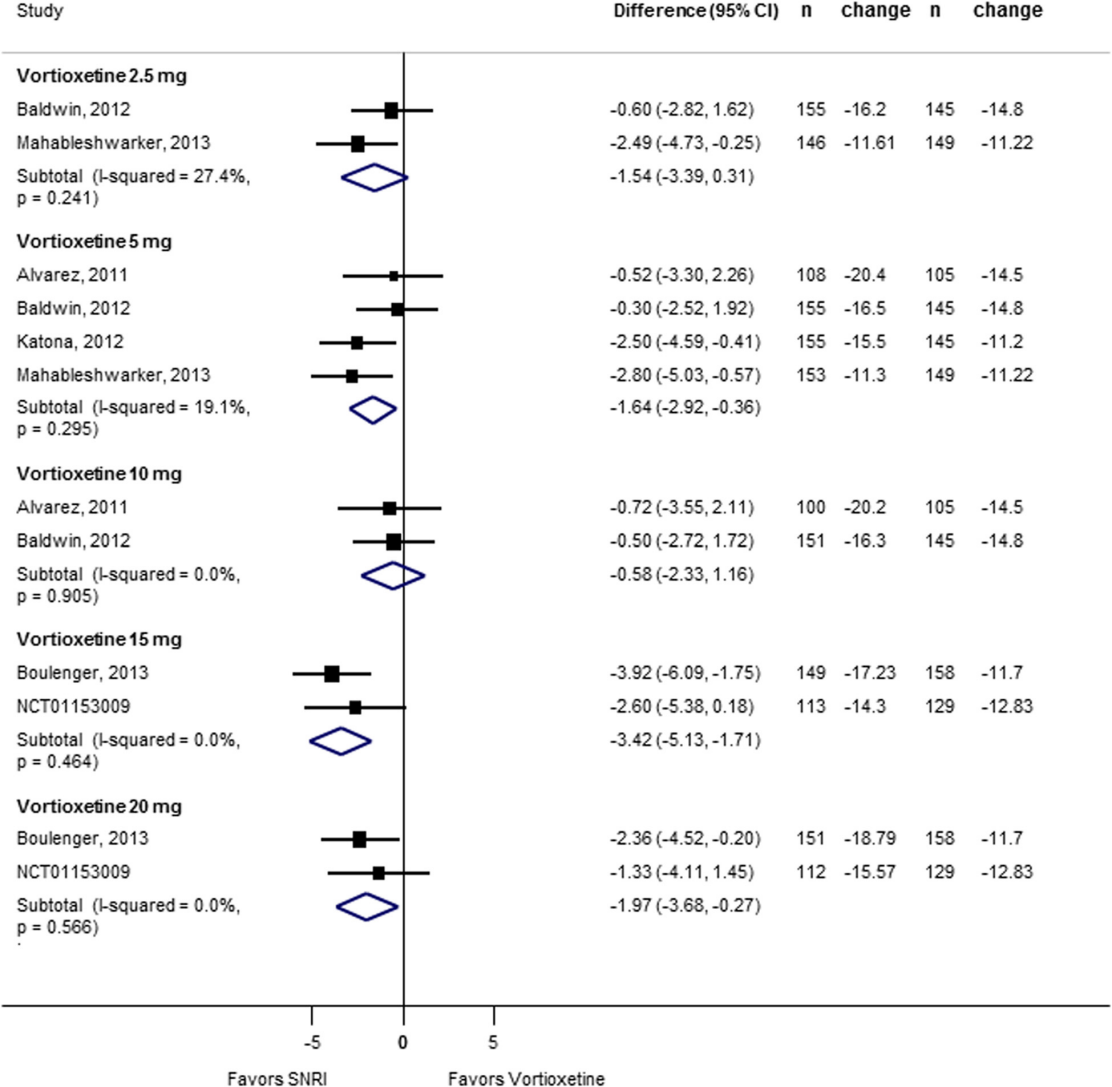
**Forest plot showing change from baseline in MADRS score for vortioxetine by dose compared to a serotonin norepinephrine reuptake inhibitor.**

As shown in Figure 6, when compared to an SNRI (venlafaxine extended release (XR) 225 mg or duloxetine 60 mg), vortioxetine response was not statistically different for the 2.5-mg (RR = 0.89; 95% CI 0.76 to 1.04; *I*^2^ = 2.5%) and 10-mg (RR = 0.98; 95% CI 0.86 to 1.11; *I*^2^ = 0%) doses. Rates of response were significantly lower than the SNRI for the 5-mg (RR = 0.88; 95% CI 0.80 to 0.98; *I*^2^ = 13%), 15-mg (RR = 0.78; 95% CI 0.68 to 0.90; *I*^2^ = 0%), and 20-mg (RR = 0.82; 95% CI 0.72 to 0.94; *I*^2^ = 0%) doses. Heterogeneity was low for all dose comparisons. Removing the study which used venlafaxine (Alvarez et al.) had no impact on treatment effect. As shown in Figure 7, no dose of vortioxetine was statistically better, and the 5-mg dose was significantly worse (RR = 0.81; 95% CI 0.66 to 0.999; *I*^2^ = 48%) than an SNRI for achieving remission; however, heterogeneity was high for several comparisons and pooled estimates may be unreliable because of the small sample size. Figure 8 shows absolute change from baseline in MADRS score for vortioxetine compared to an SNRI. Similar to response, the 5-mg (−1.64; 95% CI −2.92 to −0.36; *I*^2^ = 19%), 15-mg (−3.42; 95% CI −5.13 to −1.71; *I*^2^ = 0%), and 20-mg (−1.97; −3.68 to −0.27; *I*^2^ = 0%) doses were significantly inferior to the SNRI comparator.

### Comparative adverse events

Comparative harms for vortioxetine relative to an SNRI are summarized in Table 4. AEs generally occurred at a lower frequency with vortioxetine than venlafaxine or duloxetine. As the dose of vortioxetine was increased, differences between vortioxetine and the SNRI were reduced. At the 20-mg dose, only hyperhidrosis was significantly less common with vortioxetine. However, discontinuation due to an AE was significantly higher with vortioxetine 20 mg than the SNRI. Additional analyses are shown in Additional file 2.

**Table 4:** **Absolute risk difference of adverse events for vortioxetine compared to a serotonin norepinephrine reuptake inhibitor**

	**1 mg**	**2.5 mg**	**5 mg**	**10 mg**	**15 mg**	**20 mg**
Withdrawals due to adverse events	No trials	−6.2% (95% CI −10.7 to −1.8%)*; *I* ^2^ = 0% 2 trials	−4.7% (95% CI −9.0 to −0.4%)*; *I* ^2^ = 38% 4 trials	−4.4% (95% CI −9.7 to 0.9%); *I* ^2^ = 0% 2 trials	2.3% (95% CI −1.7 to 6.3%); *I* ^2^ = 0% 2 trials	4.4% (95% CI 0.1 to 8.7%)*; *I* ^2^ = 0% 2 trials
Serious adverse events		−1.0% (95% CI −2.5 to 0.6%); *I* ^2^ = 0% 2 trials	0.1%(95% CI −1.1 to 1.3%); *I* ^2^ = 0% 4 trials	0.4% (95% CI −1.6 to 2.4%); *I* ^2^ = 0% 2 trials	−0.3% (95% CI −3.6 to 3.1%); *I* ^2^ = 74% 2 trials	−0.1% (95% CI −1.3 to 1.1%); *I* ^2^ = 0% 2 trials
Nausea		−21.2% (95% CI −30.2 to −12.3%); *I* ^2^ = 42% 2 trials	−12.2% (95% CI −17.4 to −7.0%); *I* ^2^ = 0% 4 trials	−4.2% (95% CI −20.0 to 11.5%); *I* ^2^ = 73% 2 trials	−2.8% (95% CI −10.3 to 4.7%); *I* ^2^ = 73% 2 trials	−1.1% (95% CI −8.6 to 6.4%); *I* ^2^ = 0% 2 trials
Vomiting		−2.8% (95% CI −5.5 to −0.1%)*; *I* ^2^ = 0% 2 trials	−1.1% (95% CI −3.7 to 1.5%); *I* ^2^ = 0% 3 trials	1.2% (95% CI −6.5 to 9.0%); *I* ^2^ = 71% 2 trials	−3.2% (95% CI −8.8 to 2.3%) 1 trial	0.4% (95% CI −5.7 to 6.6%) 1 trials
Headache		−0.3% (95% CI −5.8 to 5.2%); *I* ^2^ = 0% 2 trials	−1.8% (95% CI −5.8 to 2.2%); *I* ^2^ = 0% 4 trials	−2.1% (95% CI −8.5 to 4.3%); *I* ^2^ = 0% 2 trials	−0.6% (95% CI −6.0 to 4.9%); *I* ^2^ = 0% 2 trials	−1.8% (95% CI −9.0 to 5.5%); *I* ^2^ = 43% 2 trials
Diarrhea		−3.7% (95% CI −11.8 to 4.4%); *I* ^2^ = 77% 2 trials	−1.8% (95% CI −5.0 to 1.4%); *I* ^2^ = 26% 4 trials	1.5% (95% CI −2.4 to 5.3%); *I* ^2^ = 0% 2 trials	−0.8% (95% CI −5.2 to 3.6%); *I* ^2^ = 7% 2 trials	−1.6% (95% CI −7.6 to 4.4%); *I* ^2^ = 46% 2 trials
Dizziness		−9.9% (95% CI −14.7 to −5.1%)*; *I* ^2^ = 0% 2 trials	−7.0% (95% CI −12.5 to −1.5%)*; *I* ^2^ = 62% 4 trials	−9.1% (95% CI −15.7 to −2.5%)*; *I* ^2^ = 40% 2 trials	−5.7% (95% CI −10.3 to −1.0)*; *I* ^2^ = 0% 2 trials	−4.2% (95% CI −9.0 to 0.6%); *I* ^2^ = 0% 2 trials
Dry mouth		−9.5% (95% CI −22.7 to 3.8%); *I* ^2^ = 86% 2 trials	−10.4% (95% CI −18.1 to 2.8%)*; *I* ^2^ = 77% 4 trials	−5.2% (95% CI −9.8 to −0.5%)*; *I* ^2^ = 5% 2 trials	−6.8% (95% CI −11.3 to −2.3%)*; *I* ^2^ = 0% 2 trials	−3.4% (95% CI −8.3 to 1.5%); *I* ^2^ = 0% 2 trials
Hyperhydrosis		−5.3% (95% CI −8.5 to −2.2%)*; *I* ^2^ = 0% 2 trials	−6.0% (95% CI −9.5 to −2.6%)*; *I* ^2^ = 35% 4 trials	−4.6% (95% CI −8.6 to −0.6%)*; *I* ^2^ = 0% 2 trials	−4.5% (95% CI −7.5 to −1.4%)*; *I* ^2^ = 0% 2 trials	−5.4% (95% CI −9.4 to −1.4%)*; *I* ^2^ = 43% 2 trials
Nasopharyngitis		5.8% (95% CI 1.1 to 10.5%)* 1 trial	4.6% (95% CI 1.0 to 8.3%)*; *I* ^2^ = 0% 2 trials	1.4% (95% CI −1.6 to 4.3%); *I* ^2^ = 0% 2 trials	0.7% (95% CI −3.6 to 5.0%) 1 trial	2.5% (95% CI −2.2 to 7.2%) 1 trial
Insomnia		−3.3% (95% CI −7.1 to 0.5%); *I* ^2^ = 0% 2 trials	−3.2% (95% CI −6.7 to 0.2%); *I* ^2^ = 0% 3 trials	−6.4% (95% CI −10.5 to −2.3%)*; *I* ^2^ = 0% 2 trials	−5.9% (95% CI −11.4 to −0.4%)* 1 trial	−0.9% (95% CI −7.3 to 5.5%) 1 trial
Fatigue		−5.3% (95% CI −8.3 to −2.3%)*; *I* ^2^ = 0% 2 trials	−4.7% (95% CI −7.3 to −2.1%)*; *I* ^2^ = 0% 4 trials	−3.3% (95% CI −6.9 to 0.3%); *I* ^2^ = 0% 2 trials	−3.7% (95% CI −8.8 to 1.4%); *I* ^2^ = 43% 2 trials	−3.6% (95% CI −7.6 to 0.3%); *I* ^2^ = 11% 2 trials

*P < 0.05.

### Publication bias

**Figure 9:**
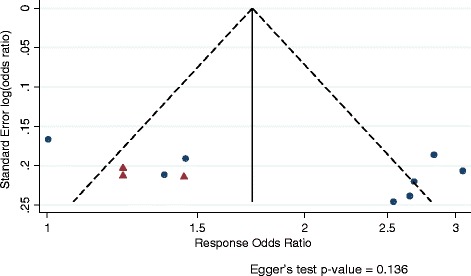
**Funnel plot of response odds ratio for vortioxetine versus placebo.** Vortioxetine dose arms combined.

**Figure 10:**
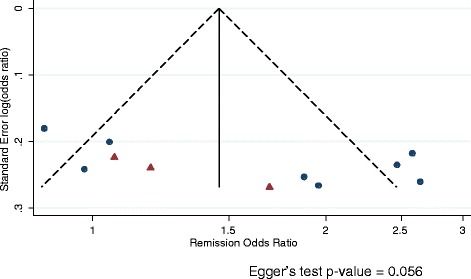
**Funnel plot of remission odds ratio for vortioxetine versus placebo.** Vortioxetine dose arms combined.

The funnel plots of response and remission shown in Figures 9 and 10 do not suggest additional unpublished trials. Egger’s tests were also not significant.

## Discussion

In this systematic review, we identified 11 studies that evaluated response, remission, and change in baseline depression scores of vortioxetine compared with placebo or an SNRI in the treatment of MDD. Three of these studies remain unpublished at the time of our analysis. Two of the three unpublished studies did not show a significant difference in response for any dosing arms compared to placebo. Pooled treatment effect estimates varied markedly between studies. There does not appear to be a dose response when compared to placebo, suggesting that doses as low as 5 mg may be as effective as doses of 20 mg.

Vortioxetine does not appear to be more effective, and is potentially less effective, than an SNRI. The SNRI comparator had significantly higher response rates compared to vortioxetine 5-, 15-, and 20-mg doses. Significant differences in MADRS change from baseline relative to an SNRI ranged from 1.64 for the 5-mg dose to 3.42 for the 15-mg dose. For remission, only the 5-mg vortioxetine dose was significantly worse than an SNRI.

In terms of safety, at the FDA-recommended target doses of 15 and 20 mg, the most common adverse effects were nausea and vomiting occurring in about 20% (number needed to harm (NNH) = 5) and 5% (NNH = 20) more patients receiving vortioxetine than placebo, respectively. The 5-mg dose of vortioxetine was only associated with an increased absolute risk of nausea and vomiting of 12% (NNH = 9) and 2% (NNH = 50), respectively. Rates of serious adverse events were similar at all dosing levels. When compared to the SNRI group, vortioxetine generally had lower rates of adverse events at the lower dose levels. At the higher doses rates, adverse events were generally similar to an SNRI.

Variables significantly associated with response were whether the study was conducted in the US and the proportion of study participants who were White. Studies with greater numbers of non-White participants (≥20%) were consistently negative and studies with lower numbers of non-White participants were consistently positive. This pattern roughly paralleled study location, where studies conducted within the US (Mahableshwarker et al., Jain et al., NCT01153009, NCT01163266, NCT01179516) all had a large (>20%) proportion of participants who were non-white and were largely negative. It is unclear why treatment effect differed by study site or racial composition. In their deliberations, the FDA recognized that efficacy of vortioxetine was generally less favorable in studies conducted in the US. In their review, the FDA deemed the 20-mg dose as the most consistently positive dosing arm among US-based trials. The other four positive trials supported the efficacy of 5-, 10-, and 15-mg doses in at least one trial, but were predominately non-US based. The final product labeling reflects this interpretation by stating “Dosage should then be increased to 20 mg/day, as tolerated, because higher doses demonstrated better treatment effects in trials conducted in the United States.” The FDA review summarized several pooled subgroup analyses of these six positive trials and found no variable to be statistically significant except region (non-US vs other) [20]. However, in their analysis of change from the baseline MADRS score, the FDA note that White participants tended to have larger treatment effects compared to Black or Asian participants. It is important to note that while the clinical trial program for vortioxetine originally consisted of ten controlled trials, only the six positive trials were extensively analyzed for the application. The remaining four negative or failed trials, which enrolled higher numbers of non-White participants (21% to 29% non-White), were not fully analyzed in the FDA’s review. As such, their exclusion from the sponsor’s individual patient level subgroup analyses exploring race is a critical deficiency, and further research is needed to understand whether there is a difference in the efficacy of vortioxetine in regards to diverse populations.

There are at least four limitations to this systematic review. First, initial meta-analyses had significant heterogeneity. Although this was resolved by subgrouping trials by racial composition, it is not clear if this variable is truly an effect modifier. Vortioxetine is primarily metabolized through the cytochrome P450 2D6 enzyme, which is known to vary between racial groups. However, the FDA clinical pharmacology review concluded that race did not have a significant impact on vortioxetine’s pharmacokinetic profile [21]. As such, the observed association between racial composition and response may be due to some other unknown patient or study site characteristic. A pharmacokinetic phase III study reviewed by EMA found that non-quantifiable samples were significantly more prevalent at US sites relative non-US sites, suggesting mediation adherence may have been a problem [22]. Because race is a known predictor of medication adherence, racial composition in our study may indicate trials where compliance was poor [23]. A second important limitation is that our analysis relied on *post hoc* standardized approaches to investigate potential reasons for heterogeneity, including sensitivity analyses and meta-regression, and therefore, their results should be interpreted with caution. The association between racial composition and efficacy cannot be fully understood by these analyses and should be studied in future work. Third, our analysis relies on aggregated summary data rather than individual patient data (IPD), like most systematic reviews. A major advantage of IPD is the ability to conduct subgroup analyses free of potential ecologic fallacy. For this study, analysis of IPD could disentangle the related issues of racial composition and geography, both of which were associated with efficacy. Finally, trials were short in duration (6 to 8 weeks) and limited to randomized controlled trials. Longer trials and non-randomized trials, which can provide important information for assessing harms, were not included in this analysis.

We purposely sought to identify and include all relevant published and unpublished trials. To accomplish this, we made use of the FDA website (Drugs@FDA.gov) and the ClinicalTrials.gov result database as alternative sources of summary trial data. The use of FDA medical and statistical officer review documents has been recognized as an important source of trial data for systematic reviews [24]. In addition to FDA documents, we also used summary results posted to ClinicalTrials.gov. Data summarized in ClinicalTrials.gov were critical to incorporating unpublished trial data as well as filling in the gaps for outcomes that were either not reported or reported ambiguously in the publication. ClinicalTrials.gov was particularly useful for negative or failed trials, because these trials are only briefly summarized by the FDA [25]. Also, there was one trial in our review that was not at all considered by the FDA [26]. Although ClinicalTrials.gov result summaries have the potential to be a great resource for complimenting systematic reviews, questions about its ultimate validity remain [27-29]. With the inclusion of unpublished studies, our funnel plot analyses did not suggest the presence of other potentially unpublished trials. However, with only 11 total trials, the power of these analyses was limited [30].

## Conclusions

Similar to the FDA, we found that vortioxetine was significantly more effective in response and remission than placebo for acute treatment of MDD. However, our study suggests that vortioxetine may not be more effective, and is potentially less effective, than an SNRI. We found no evidence of a dose effect for vortioxetine with the exception of adverse effects. The 20-mg dose was approved as the target dose by the FDA because it was the only dose with at least two trials showing efficacy in the US population. Our exploratory observation that studies with higher non-white racial composition were less likely to respond requires further study.

## Additional files

Additional file 1:
**Individual trial data with quality ratings.**
Additional file 2:
**Additional meta-analysis figures and tables.**

## Abbreviations

5-HT: serotonin
95% CI: 95% confidence interval
AE: adverse event
DERP: Drug Effectiveness Review Project
EMA: European Medicines Agency
FDA: Food and Drug Administration
HAMD: Hamilton Depression Rating Scale
IPD: individual patient data
MADRS: Montgomery-Åsberg Depression Rating Scale
MDD: major depressive disorder
NNH: number needed to harm
PRISMA: Preferred Reporting Items for Systematic Reviews and Meta-Analyses
RCT: randomized controlled trial
RR: relative risk
SNRI: serotonin and norepinephrine reuptake inhibitor
SSRI: selective serotonin reuptake inhibitor
US: United States
XR: extended release
