# Supplementary material for: The safety and efficacy of vortioxetine for acute treatment of major depressive disorder: a systematic review and meta-analysis
Source: Syst Rev. 2015 Mar 1;4:21. doi: 10.1186/s13643-015-0001-y (PMC4351824; doi:10.1186/s13643-015-0001-y)
Supplement: Additional file 2: — Additional meta-analysis figures and tables. [file 13643_2015_1_MOESM2_ESM.doc]

Appendix 2: Forest plot showing change from baseline in MADRS total score for vortioxetine by dose compared to placebo


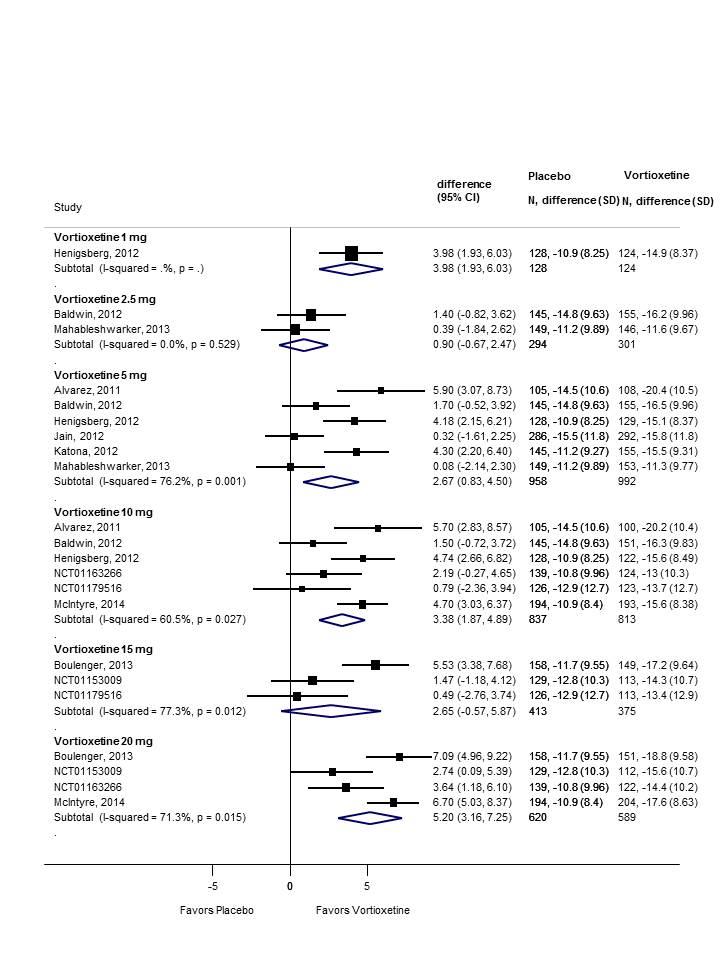


Appendix 3: Meta-regression of study characteristics on log odds ratio for response.

| **Variable** | **Coefficient** | **95% Confidence Interval** | **p-value** | **Residual I^2^** |
| --- | --- | --- | --- | --- |
| Dose* | 0.01 | -0.02 to 0.04 | 0.46 | 67% |
| Unpublished | -0.44 | -1.04 to 0.16 | 0.13 | 74% |
| Poor quality | 0.44 | -0.57 to 1.45 | 0.35 | 77% |
| Non-US based study | -0.70 | -1.02 to -0.35 | 0.001 | 24% |
| Duration 8 weeks (vs 6 weeks) | 0.10 | -0.29 to 0.48 | 0.58 | 77% |
| Baseline MADRS score | -0.11 | -0.33 to 0.11 | 0.29 | 75% |
| Proportion of study participants non-White | -0.04 | -0.05 to -0.02 | <0.001 | 0% |

* Placebo arm partitioned for multi-arm tri

Appendix 4: Summary of response and remission risk differences and 95% confidence intervals (CI) of vortioxetine versus placebo by dose according to race (>20% non-White participants, <20% non-White participants)

|  | Study Population >20% non-White | | | Study Population < 20% non-White | | |
| --- | --- | --- | --- | --- | --- | --- |
|  | Response | Remission | Change in MADRS Total Score | Response | Remission | Change in MADRS Total Score |
| 1 mg |  |  |  | 1.91  (1.36 to 2.69)  1 trial | 1.57  (0.98 to 2.50)  1 trial | -3.98  (-6.04 to -1.92)  1 trial |
| 2.5 mg | 1.20  (1.00 to 1.43); I^2^=0%  2 trials | 0.99  (0.77 to 1.28); I^2^=0%  2 trials | -1.40  (-0.90 to 0.68); I^2^=0%  2 trials |  |  |  |
| 5 mg | 1.09 (0.96 to 1.24); I^2^=0%  3 trials | 0.96 (0.81 to 1.15)  3 trials, I^2^=0% | -0.66 (-1.88 to 0.56)  3 trials, I^2^=0% | 1.62 (1.38 to 2.15); I^2^=0%  3 trials | 1.74  (1.38 to 2.19);  I2=0%  3 trials | -4.59  (-5.89 to -3.28);  I2=0%  3 trials |
| 10 mg | 1.21  (1.03 to 1.41); I^2^=0%  3 trials | 1.18  (0.95 to 1.47); I^2^=0%  3 trials | -1.50  (-3.06 to -0.13); I^2^=0%  3 trials | 1.67  (1.42 to 1.96); I^2^=0%  3 trials | 1.76  (1.40 to 2.22); I^2^=12%  3 trials | -4.96  (-6.38 to -3.54);  I2=0%  3 trials |
| 15 mg | 1.13  (0.92 to 1.39); I^2^=0%  2 trials | 1.04  (0.78 to 1.37); I^2^=0%  2 trials | -1.08  (-3.14 to 0.99); I^2^=0%  2 trials | 1.77  (1.36 to 2.30)  1 trial | 1.84  (1.24 to 2.71)  1 trial | -5.53  (-7.69 to -3.37)  1 trial |
| 20 mg | 1.23  (1.00 to 1.51); I^2^=0%  2 trials | 1.09  (0.89 to 1.79); I^2^=0%  2 trials | -3.23  (-5.03 to -1.42); I^2^=0%  2 trials | 1.91  (1.65 to 2.35); I^2^=0%  2 trials | 2.15  (1.66 to 2.79); I^2^=0%  2 trials | -7.09  (-8.85 to -5.14);  I2=0%  3 trials |

Appendix 5: Forest plot showing change from baseline in MADRS score for vortioxetine by dose compared to a serotonin norepinephrine reuptake inhibitor


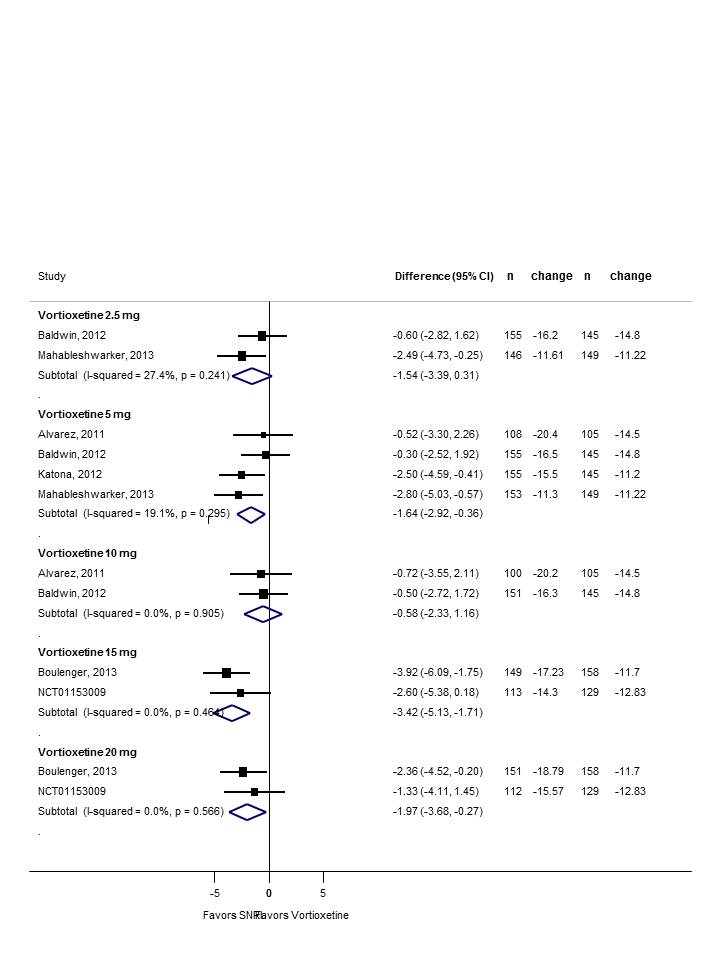


|  | 1 mg | 2.5 mg | 5 mg | 10 mg | 15 mg | 20 mg |
| --- | --- | --- | --- | --- | --- | --- |
| Withdrawals due to adverse events | No trials | -6.2% (95% CI -10.7% to -1.8%)*; I^2^=0% 2 trials | -4.7% (95% CI -9.0% to -0.4%)*; I^2^=38% 4 trials | -4.4% (95% CI -9.7% to 0.9%); I^2^=0% 2 trials | 2.3% (95% CI -1.7% to 6.3%); I^2^=0% 2 trials | 4.4% (95% CI 0.1% to 8.7%)*; I^2^=0% 2 trials |
| Serious adverse events |  | -1.0% (95% CI -2.5% to 0.6%); I^2^=0% 2 trials | 0.1%(95% CI -1.1% to 1.3%); I^2^=0% 4 trials | 0.4% (95% CI -1.6% to 2.4%); I^2^=0% 2 trials | -0.3% (95% CI -3.6% to 3.1%); I^2^=74% 2 trials | -0.1% (95% CI -1.3% to 1.1%); I^2^=0% 2 trials |
|  |  |  |  |  |  |  |
| Nausea |  | -21.2% (95% CI -30.2% to -12.3%); I^2^=42% 2 trials | -12.2% (95% CI -17.4% to -7.0%); I^2^=0% 4 trials | -4.2% (95% CI -20.0 to 11.5%); I^2^=% 2 trials | -2.8% (95% CI -10.3% to 4.7%); I^2^=73% 2 trials | -1.1% (95% CI -8.6% to 6.4%); I^2^=0% 2 trials |
| Vomiting |  | -2.8% (95% CI -5.5% to -0.1%)*; I^2^=0% 2 trials | -1.1% (95% CI -3.7% to 1.5%); I^2^=0% 3 trials | 1.2% (95% CI -6.5% to 9.0%); I^2^=71% 2 trials | -3.2% (95% CI -8.8% to 2.3%) 1 trial | 0.4% (95% CI -5.7% to 6.6%) 1 trials |
| Headache |  | -0.3% (95% CI -5.8% to 5.2%); I^2^=0% 2 trials | -1.8% (95% CI -5.8% to 2.2%); I^2^=0% 4 trials | -2.1% (95% CI -8.5% to 4.3%); I^2^=0% 2 trials | -0.6% (95% CI -6.0% to 4.9%); I^2^=0% 2 trials | -1.8% (95% CI -9.0% to 5.5%); I^2^=43% 2 trials |
| Diarrhea |  | -3.7% (95% CI -11.8% to 4.4%); I^2^=77% 2 trials | -1.8% (95% CI -5.0% to 1.4%); I^2^=26% 4 trials | 1.5% (95% CI -2.4% to 5.3%); I^2^=0% 2 trials | -0.8% (95% CI -5.2% to 3.6%); I^2^=7% 2 trials | -1.6% (95% CI -7.6% to 4.4%); I^2^=46% 2 trials |
| Dizziness |  | -9.9% (95% CI -14.7% to -5.1%)*; I^2^=0% 2 trials | -7.0% (95% CI -12.5% to  -1.5%)*; I^2^=62% 4 trials | -9.1% (95% CI -15.7% to -2.5%)*; I^2^=40% 2 trials | -5.7% (95% CI -10.3% to -1.0)*; I^2^=0% 2 trials | -4.2% (95% CI -9.0% to 0.6%); I^2^=0% 2 trials |
| Dry mouth |  | -9.5% (95% CI -22.7% to 3.8%); I^2^=86% 2 trials | -10.4% (95% CI  -18.1% to 2.8%)*; I^2^=77% 4 trials | -5.2% (95% CI -9.8% to -0.5%)*; I^2^=5% 2 trials | -6.8% (95% CI -11.3 to -2.3%)*; I^2^=0% 2 trials | -3.4% (95% CI -8.3% to 1.5%); I^2^=0% 2 trials |
| Hyperhydrosis |  | -5.3% (95% CI -8.5% to -2.2%)*; I^2^=0% 2 trials | -6.0% (95% CI -9.5% to -2.6%)*; I^2^=35% 4 trials | -4.6% (95% CI -8.6% to -0.6%)*; I^2^=0% 2 trials | -4.5% (95% CI -7.5% to -1.4%)*; I^2^=0% 2 trials | -5.4% (95% CI -9.4% to -1.4%)*; I^2^=43% 2 trials |
| Nasopharyngitis |  | 5.8% (95% CI 1.1% to 10.5%)* 1 trial | 4.6% (95% CI 1.0% to 8.3%)*; I^2^=0% 2 trials | 1.4% (95% CI-1.6% to 4.3%); I^2^=0% 2 trials | 0.7% (95% CI -3.6% to 5.0%) 1 trial | 2.5% (95% CI -2.2% to 7.2%) 1 trial |
| Insomnia |  | -3.3% (95% CI -7.1% to 0.5%); I^2^=0% 2 trials | -3.2% (95% CI -6.7% to 0.2%); I^2^=0% 3 trials | -6.4% (95% CI -10.5% to -2.3%)*; I^2^=0% 2 trials | -5.9% (95% CI -11.4% to -0.4%)* 1 trial | -0.9% (95% CI -7.3% to 5.5%) 1 trial |
| Fatigue |  | -5.3% (95% CI -8.3% to -2.3%)*; I^2^=0% 2 trials | -4.7% (95% CI -7.3% to -2.1%)*; I^2^=0% 4 trials | -3.3% (95% CI -6.9% to 0.3%); I^2^=0% 2 trials | -3.7% (95% CI -8.8% to 1.4%); I^2^=43% 2 trials | -3.6% (95% CI -7.6% to 0.3%); I^2^=11% 2 trials |

Appendix 6: Absolute risk difference of adverse events for vortioxetine compared to a serotonin norepinephrine reuptake inhibitor. *p<0.05
